# Investigation into the impact of CeO_2_ morphology regulation on the oxidation process of dichloromethane

**DOI:** 10.1039/d4ra01326c

**Published:** 2024-04-16

**Authors:** Hao Wu, Xiaoliang Zhao, Jian Li, Bandna Bharti, Yuling Tan, Hongyan Long, Jiuhu Zhao, Gang Tian, Fan Wang

**Affiliations:** a Institute of Atmospheric Environment, Chinese Research Academy of Environmental Sciences Beijing 100012 China fanwangsd@163.com; b College of Environmental Science and Engineering, Liaoning Technical University Fuxin 123000 China; c Department of Chemistry, DAV University Jalandhar Punjab 144001 India; d Harbin Institute of Technology Shenzhen Shenzhen 518055 China

## Abstract

Four distinct CeO_2_ catalysts featuring varied morphologies (nanorods, nanocubes, nanoparticles, and nano spindle-shaped) were synthesized through a hydrothermal process and subsequently employed in the oxidation of dichloromethane (DCM). The findings revealed that the nano spindle-shaped CeO_2_ exhibited exposure of crystal faces (111), demonstrating superior catalytic oxidation performance for DCM with a *T*_90_ of 337 °C and notably excellent low-temperature catalytic activity (*T*_50_ = 192 °C). The primary reaction products were identified as HCl and CO_2_. Through obvious characterizations, it showed that the excellent catalytic activity presented by CeO_2_-s catalyst might be related to the higher oxygen vacancy concentration, surface active oxygen content, and superior redox performance caused by specific exposed crystal planes. Meanwhile, CeO_2_-s catalyst owned outstanding stability, reusability, and water inactivation regeneration, which had tremendous potential in practical treatment.

## Abstract 2

Four distinct CeO_2_ catalysts featuring varied morphologies (nanorods, nanocubes, nanoparticles, and nano spindle-shaped) were synthesized through a hydrothermal process and subsequently employed in the oxidation of dichloromethane (DCM).

## Graphical abstract

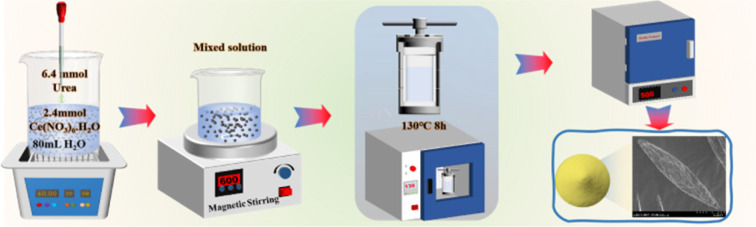

## 1 Introduction

Volatile Organic Compounds (VOCs) possess a multifaceted composition, undergoing decomposition upon exposure to light, thereby generating free radicals and peroxyl radicals. These radicals serve as pivotal precursors for the production of ozone and fine particulate matter, posing a substantial threat to the environment.^1^ Concurrently, VOCs are known to inflict severe damage on the human respiratory, nervous, and immune systems.^2,3^ Notably, chlorinated volatile organic compounds (CVOCs) are characterized by high toxicity, volatility, and resistance to degradation, emerging as significant pollutants with detrimental impacts on ecological ecosystems and human health.^4^ DCM, a representative CVOCs, finds widespread application as an organic solvent in sectors such as pharmaceuticals, spray coating, and rubber manufacturing.^5^ Urgency surrounds the imperative to fortify DCM treatment strategies. Common approaches for conducting the treatment of CVOCs encompass adsorption, absorption, condensation, combustion, low-temperature plasma, and catalytic oxidation.^6,7^ Catalytic oxidation, propelled by a catalyst, efficiently and comprehensively converts CVOCs into relatively non-toxic substances, including H_2_O, CO_2_, and HCl, with minimal energy consumption. It has evolved into the predominant technology applied in the CVOCs treatment sector in both China and internationally.^8^ Consequently, the preparation of catalysts that are efficient, stable, and cost-effective assumes paramount significance in enhancing the competitiveness of this technology. While precious metal catalysts boast merits such as a low ignition temperature, high activity, and elevated HCl selectivity, their scarcity contributes to prohibitively high costs. Furthermore, the vulnerability of catalyst surfaces composed of precious metals to carbon deposition, coupled with the adsorption of chlorine from CVOCs onto active sites, leads to the phenomenon of chlorine poisoning, ultimately culminating in catalyst deactivation subsequent to chlorine deposition.^9,10^ In recent years, non-precious metal catalysts have garnered extensive scholarly attention in China and globally due to their relatively high activity, cost-effectiveness, and resilience against chlorine poisoning.

In addition, nanomaterials have unique physical, chemical and biological properties that make them promising for a wide range of applications in catalysis. Atul S. Nagpure *et al.*^11^ studied the catalytic transfer hydrogenation of 5-hydroxymethylfurfural (HMF) to 2,5-dimethylfuran (DMF) and furfural to 2-methylfuran (MF) using 2-propanol as hydrogen source on nitrogen-doped mesoporous carbon (NMCs) supported Ru, Pd and Au metal catalysts. It was shown that highly dispersed Ru nanoparticles loaded on NMC exhibited excellent catalytic performance for the conversion of HMF to DMF and furfural to MF in the CTH reaction. This is mainly attributed to the smaller nanoparticle size of Ru (1.9 nm) and the good interaction between the metal and the carrier. Zhu *et al.*^12^ used Cs_3_Sb_2_Br_9_ perovskite nanoparticles (NPs) as a lead-free photocatalysts for photocontrolled atom transfer radical polymerisation (ATRP). Cs_3_Sb_2_Br_9_ NPs have a high reduction potential, which enables efficient photo-induced reduction and controlls polymerisation of the initiator under blue light irradiation. Meanwhile, the Cs_3_Sb_2_Br_9_ NPs can be recycled four times, showing good reusability.

China boasts abundant reserves of rare earth elements, establishing its position as the leading global rare earth supplier, contributing to over 90 percent of the world's total rare earth production annually. These rare earth elements find extensive utilization in various applications, encompassing magnetic materials, luminescent materials, and catalysts dedicated to environmental protection.^13^ Among the rare earth materials, cerium dioxide (CeO_2_) assumes a pivotal role due to its inherent attributes, including a stable cubic fluorite structure. Notably, the facile interconversion between Ce^3+^ and Ce^4+^ ions endows CeO_2_ with remarkable oxygen storage and release capabilities and a pronounced redox performance. Concurrently, this interconversion results in the creation of oxygen vacancies within the original lattice. Oxygen vacancies serve a dual function: firstly, they possess the capacity to adsorb gaseous-phase oxygen and transform it into surface-active oxygen species, and secondly, they serve as active sites for the direct adsorption of chlorinated volatile organic compounds (CVOCs), thereby enhancing catalytic efficiency. Consequently, CeO_2_ exhibits a distinct advantage in the catalytic oxidation of CVOCs.^14–16^ Furthermore, in addition to the intrinsic properties of CeO_2_ delineated previously, the manipulation of CeO_2_'s morphology exerts a significant impact on both the selectivity towards exposed crystal faces and its redox performance capacity. This, in turn, exerts a profound impact on the catalytic oxidation performance.

Tian *et al.*^10^ employed a one-step hydrothermal method to meticulously regulate the morphology of CeO_2_, specifically targeting the exposure of distinct crystal faces. Their investigation revealed that, in comparison to alternative CeO_2_ morphologies, nano-spherical CeO_2_, exposing the (111) crystal face, exhibited exceptional redox properties, lattice oxygen mobility, and emerged as the optimal catalyst for dichloroethane catalytic oxidation. In a similar vein, Hu *et al.*^17^ manipulated CeO_2_ morphology to modulate its selectivity towards exposed crystal faces. Their findings indicated that rod-shaped CeO_2_, selectively exposing (110) and (100) crystal faces, displayed heightened mobility of reactive oxygen species, lower energy for oxygen vacancy generation, and superior catalytic oxidation performance for propane when contrasted with other morphologies. In light of these observations, this study endeavors to explore the modulation of CeO_2_ selectivity towards exposed crystal faces through the manipulation of its morphology, thereby enhancing its catalytic efficacy on pollutants. Leveraging the hydrothermal synthesis method, four distinct nanomorphologies of CeO_2_ were prepared, with DCM, an industrially prevalent compound, chosen as the subject of investigation. Conspicuously, nano spindle-shaped CeO_2_ (CeO_2_-s) has not been used for catalytic oxidation of DCM. This paper meticulously examines and analyzes the impact of varied selectivities of CeO_2_, attributed to different nanomorphologies exposing crystal faces, on DCM catalytic performance, product selectivity, stability, reusability, and water resistance. Simultaneously, the catalyst undergoes rigorous physical and chemical characterization, encompassing morphology, crystal structure, specific surface area, average pore size, surface element valence and content, oxygen vacancy concentration, and redox performance. The synthesis of this comprehensive analysis constitutes an initial step in laying the foundation for the design and advancement of efficient catalysts specifically crafted for the mitigation of Chlorinated Volatile Organic Compound (CVOC) pollutants.

## 2 Experiment

### 2.1 Catalyst preparation

**Fig. 1:**
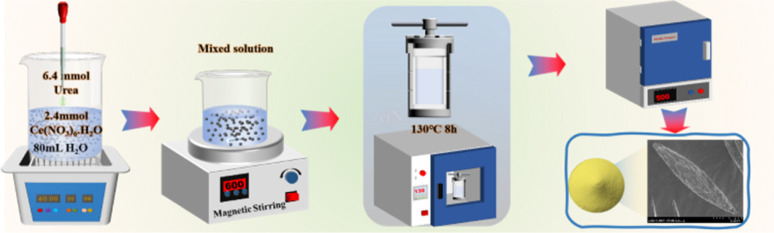
Preparation of catalyst.

The synthesis of catalyst is shown in Fig. 1. The synthesis of nano rod-shaped CeO_2_ (CeO_2_-r) involved the following sequential steps: 2.17 g of Ce(NO_3_)_3_·6H_2_O solid was dissolved in 20 mL of deionized water, followed by the gradual addition of a pre-prepared 60 mL, 6 mol L^−1^ NaOH solution to the Ce(NO_3_)_3_·6H_2_O solution using a rubber-tipped dropper. The resulting mixture was stirred at room temperature on a magnetic stirrer for 0.5 h and subsequently transferred to a 100 mL Teflon high-pressure hydrothermal autoclave for hydrothermal synthesis at 125 °C for 24 h. The synthesized product was then filtered, dried, and subjected to a final roasting step in a muffle furnace at 500 °C for 3 h, yielding pale yellow rod-like CeO_2_ powders. For the preparation of nano cube-shaped CeO_2_ (CeO_2_-c), the same procedure as CeO_2_-r was followed, with the exception that the hydrothermal synthesis temperature was adjusted to 185 °C. The synthesis of nano particle-shaped CeO_2_ (CeO_2_-p) involved the addition of 5 mmol of Ce(NO_3_)_3_·6H_2_O and 40 mmol of urea to 80 mL of deionized water. After thorough mixing, the solution was placed in a 100 mL Teflon high-pressure hydrothermal autoclave for hydrothermal synthesis at 180 °C for 10 h. Following natural cooling to room temperature, the product was filtered, dried, and subjected to a final roasting step at 500 °C for 3 h to obtain particle-shaped CeO_2_ powders. The preparation of nano spindle-shaped CeO_2_ (CeO_2_-s) commenced with the addition of 2.4 mmol of Ce(NO_3_)_3_·6H_2_O to 80 mL of deionized water in a microwave ultrasound instrument. Simultaneously, 6.4 mmol of urea was swiftly introduced to the cerium-containing solution and subjected to ultrasound concussion for 0.5 h. The resulting mixture was transferred to a magnetic stirrer, stirred at room temperature for 0.5 h, and then placed in a 100 mL Teflon high-pressure hydrothermal autoclave at 130 °C for hydrothermal synthesis for 8 h. After cooling to room temperature, the product was centrifuged, filtered, and dried. The precursor powder was further calcined at 500 °C in a muffle furnace for 3 h to obtain spindle-shaped CeO_2_ powders.

In summary, the total four different morphologies of CeO_2_ need to be heated up from room temperature to 500 °C in a muffle furnace at a rate of 2 °C min^−1^, and roasted at this temperature for 3 h. In this study, the different morphologies of CeO_2_ were prepared by hydrothermal synthesis, and the morphology of CeO_2_ was regulated by changing the hydrothermal temperature and time as well as the alkali concentration. Torrente-Murciano *et al.*^18^ conducted a careful study of the conditions for the formation of cerium oxide nanomorphology and found that both the temperature and the concentration of alkali had a significant effect on the CeO_2_ morphology. In particular, lower hydrothermal temperatures favoured the synthesis of nanoparticulate CeO_2_, while higher temperatures made it easier to synthesize nanocubic CeO_2_. Liao *et al.*^19^ prepared rod-shaped CeO_2_ by ultrasound-assisted hydrothermal method, and found that cerium precursor, alkali concentration, and ultrasound were the critical to the formation of CeO_2_ nanorods.

### 2.2 Catalyst characterization

Scanning electron microscopy (SEM) images of various CeO_2_ morphologies were acquired using a SU-8020 Scanning Electron Microscope Operating at 30 kV.

Transmission electron microscopy (TEM) images, providing insights into the microscopic morphology and exposed crystal surfaces of the samples, were obtained using a JEM-2100HR Transmission Electron Microscope.

For the assessment of crystal structure, a Bruker D8 Advance X-ray powder diffractometer was employed. The analysis utilized a Cu Kα target with a wavelength (*λ*) of 0.154058 nm, operating at 40 kV and 200 mA. The scanning parameters included a speed of 10° min^−1^, and a scanning range spanning 2*θ* = 10–80°.

The N_2_ adsorption–desorption isotherm curve was generated at 77 K using the ASAP 2020 M automatic surface analyzer, a product of the US-based Micromeritics company. The specific surface area was determined employing the Brunauer–Emmett–Teller (BET) method, and the pore size distribution was analyzed using the Barrett–Joyner–Halenda (BJH) method.

Photoelectron spectroscopy (XPS) of the CeO_2_ samples was conducted utilizing the Thermo Scientific Escalab 250 Xi X-ray photoelectron spectrometer. The C 1s calibration binding energy was established at 284.8 eV.

Raman spectroscopic analysis (Raman) was executed employing the LabRAM Aramis Raman spectrometer from HYJ, France. The excitation light source had a wavelength of 325 nm (ultraviolet), and the scanning range spanned from 200 to 1400 cm^−1^.

The temperature programmed reduction (H_2_-TPR) test was performed using the Auto Chem II 2920 chemisorption instrument. The sample underwent a temperature ramp from room temperature to 300 °C in a nitrogen (N_2_) atmosphere (30 mL min^−1^) for 1 hour, followed by a return to room temperature. Subsequently, a 5% H_2_/Ar mixture was introduced as the reducing gas, and the sample temperature was elevated from room temperature to 800 °C at a heating rate of 10 °C min^−1^.

### 2.3 Catalytic activity

**Fig. 2:**
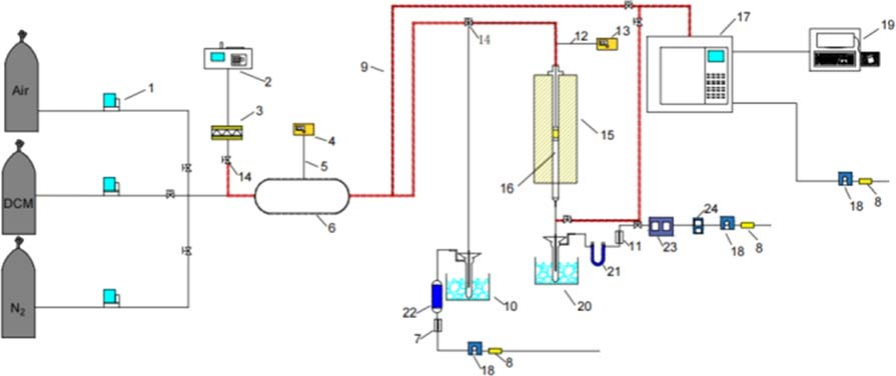
Catalyst activity evaluation device diagram. (1) Mass flow meter; (2) syringe pump; (3) carburetor; (4) temperature controller; (5) thermocouples; (6) static mixer; (7) rotor flowmeter; (8) activated carbon adsorption tube; (9) heating belt with heat tracing insulation; (10) cold trap; (11) rotor flowmeter; (12) thermocouples; (13) temperature controller; (14) valves; (15) heating furnace; (16) quartz tube; (17) gas chromatograph; (18) peristaltic pump; (19) computer; (20) cold trap; (21) U tube; (22) drier tube; (23) CO_2_ detector and CO detector; (24) chlorine gas detector.

The catalyst activity evaluation device is shown in Fig. 2. A cylindrical silica glass tube, possessing an inner diameter of 12 mm, served as the immobile reactor bed for assessing the catalytic oxidation efficiency of the prepared catalyst towards DCM. A quantity of 0.6 g of the catalyst sample (40–60 mesh) was carefully positioned within a quartz glass tube, affixed both above and below using an appropriate amount of passivated quartz wool. Subsequently, the reactor temperature underwent an incremental rise from room temperature to 150 °C, employing a heating rate of 5 °C min^−1^, with a continuous flow of nitrogen set at 200 mL min^−1^. The temperature was sustained for 0.5 h to mitigate the influence of water vapor and other impurities on the experimental outcomes. A consistent concentration of DCM, incorporated into a gas mixture (20 vol% O_2_, N_2_ as the equilibrium gas), was introduced into the system at a total gas flow rate of 200 mL min^−1^. The tubular furnace initiated a programmed temperature ascent from 150 °C to 450 °C at a rate of 5 °C min^−1^. Samples were extracted at predefined temperature intervals for subsequent analysis. The catalyzed gas underwent bifurcation for analysis. One portion, subsequent to condensation and desiccation, traversed through the CO infrared detector (SGA-700B-CO) and CO_2_ infrared detector (SGA-700B-CO_2_) to quantify concentrations of CO and CO_2_. Subsequently, it was directed towards the portable chlorine-containing gas detector to ascertain concentrations of HCl and Cl_2_. The remaining portion was introduced into the gas chromatography system (GC-7890A) to identify organic components within the reaction gas. The GC was equipped with an FID detector and an HP series capillary chromatographic column.

The calculations for DCM conversion rate and the yields of HCl, Cl_2_, CO_2_, and CO are delineated as follows:1
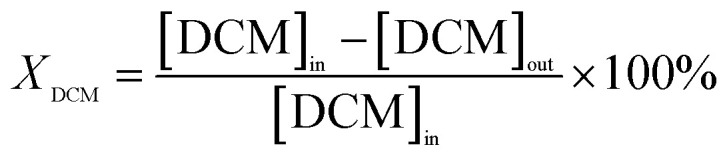
2
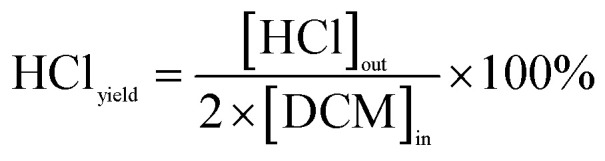
3
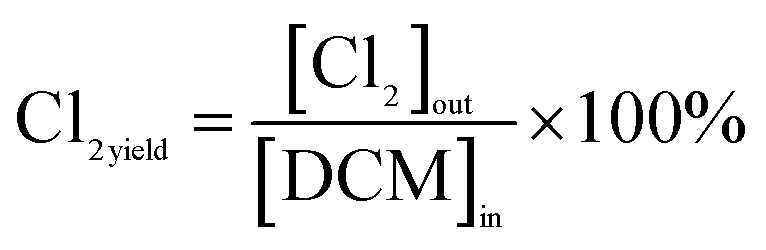
4
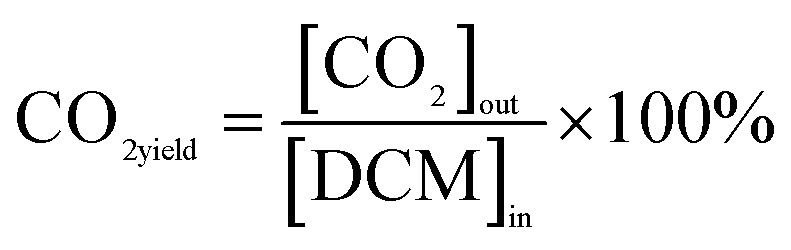
5
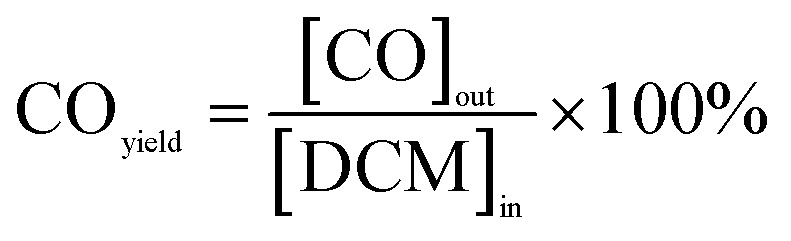
wherein, [DCM]_in_ and [DCM]_out_ denote the DCM concentration at the reactor inlet and outlet, respectively. Similarly, [HCl]_out_ signifies the hydrogen chloride concentration at the outlet, [Cl_2_]_out_ represents the chlorine concentration at the outlet, [CO_2_]_out_ indicates the carbon dioxide concentration at the outlet, and [CO]_out_ denotes the carbon monoxide concentration at the outlet.

The reaction rates for different morphologies of CeO_2_ were compared and calculated as follows:
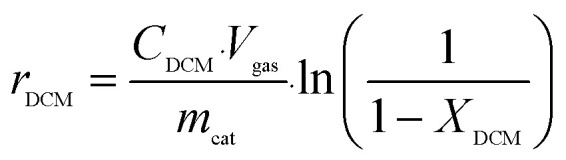
wherein, *C*_DCM_ denotes the initial concentration of DCM. *V*_gas_ is the total flow rate of the reaction gas. *m*_cat_ represents the mass of catalyst in the bed. *X*_DCM_ is the conversion rate of DCM.

## 3 Results and discussion

### 3.1 Morphology and microstructure of CeO_2_ catalysts

**Fig. 3:**
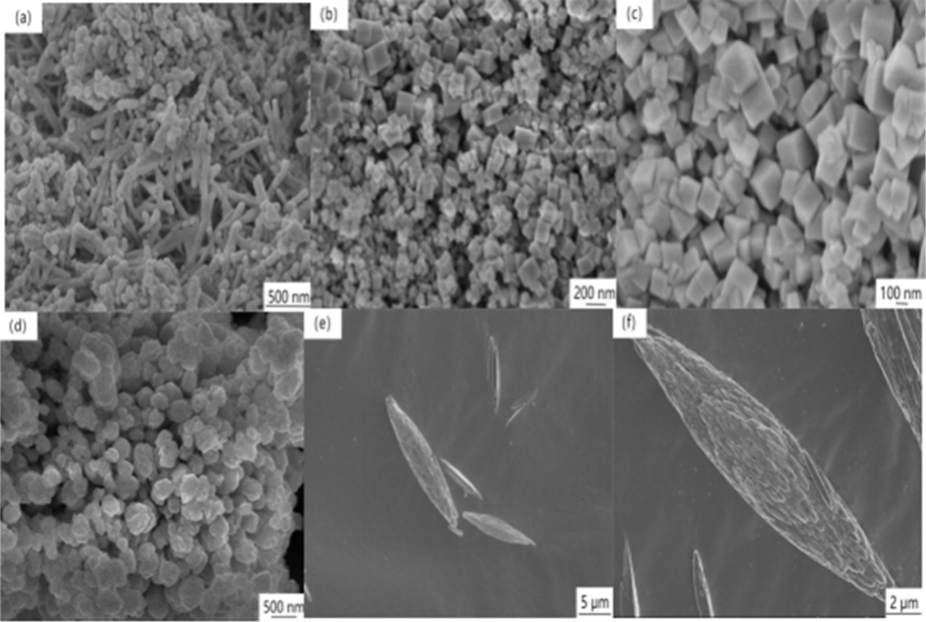
SEM of CeO_2_ with different nanomorphologies ((a) CeO_2_-r; (b and c) CeO_2_-c (d) CeO_2_-p; (e and f) CeO_2_-s).

The SEM was employed to scrutinize the morphological characteristics of the four CeO_2_ catalysts. Fig. 3(a–f) distinctly illustrates randomly dispersed rods, angular cubes, centrally located particles, and conspicuous spindle-shaped CeO_2_, respectively, aligning seamlessly with the envisioned design. Simultaneously, all catalyst samples exhibited a uniformly homogeneous morphology within a specified field of view, underscoring the consistent morphological integrity. In Fig. 3(a), CeO_2_-r was comprised of multiple nanorods with lengths of 40–350 nm. This morphology reveals inherent grain size heterogeneity, and a vertical observation of the nanorods indicates a solid structural configuration. Fig. 3(b and c), CeO_2_-c comprises numerous nanocubes, with ranging from 25–155 nm of side lengths. The smooth and angular surfaces of these nanocubes denote a high degree of crystallinity and a well-defined crystal structure.^20^Fig. 3(d) illustrates CeO_2_-p, characterized by individual spherical nanoparticles with diameters falling within the range of 60–100 nm. The distribution of these nanoparticles appears more concentrated, with slight agglomeration. Moving on to Fig. 3(e), CeO_2_-s is depicted with multiple nanospindles, with significantly larger size compared to other morphologies. These spindles exhibit lengths ranging from 4.5–10 μm and widths ranging from 1–2 μm. Upon closer examination of a single nanospindle at relatively high resolution, as depicted in Fig. 3(f), it becomes evident that the two ends of the nanospindles are relatively sharp, with curved edges differing from the smooth surface of the nanocube. Moreover, the surface of the nanospindle reveals a profusion of slits, indicating the presence of abundant defective sites. The nanospindle comprises several closely stacked nano-strips, affirming a non-monocrystalline structure. This tightly stacked framework is prone to inducing planar staggering and generating structural defects.^21^

**Fig. 4:**
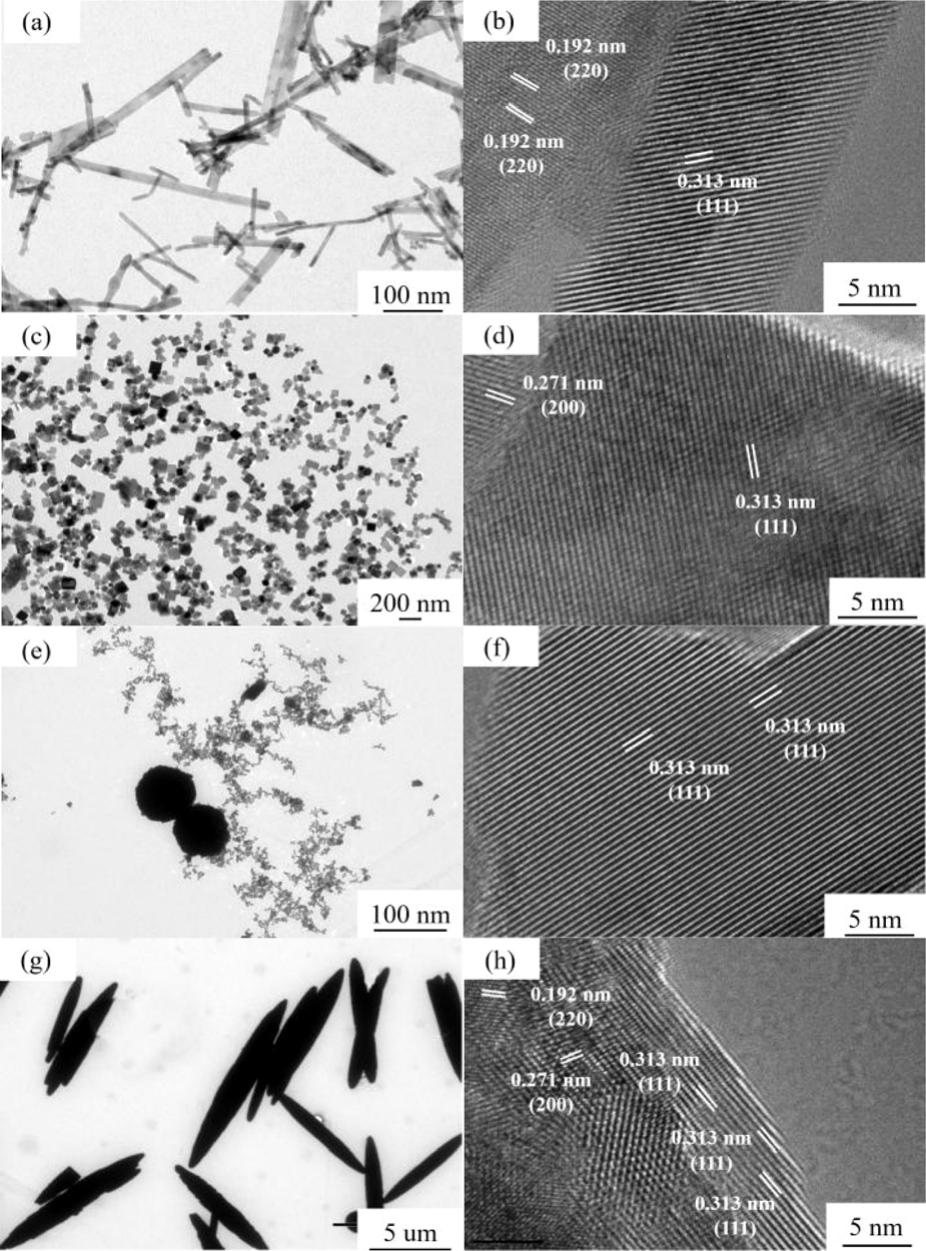
HR-TEM of CeO_2_ with different nanomorphologies ((a and b) CeO_2_-r; (c and d) CeO_2_-c; (e and f) CeO_2_-p; (g and h) CeO_2_-s).

As depicted in Fig. 4(a, c, e and g), the rod-shaped, cubic, particle, and spindle-shaped morphologies of CeO_2_ are distinctly evident. Notably, each morphology exclusively appears within the visible range, providing additional evidence of the uniformity in morphology across all catalyst samples. Furthermore, the size results of the four distinct CeO_2_ catalysts align consistently with the SEM findings. For nano rod-shaped CeO_2_, the lattice fringe spacing predominantly measures 0.192 nm, corresponding to the (110) exposed crystal faces (Fig. 4(b)). Similarly, nano cube-shaped CeO_2_ exhibits lattice spacing primarily at 0.271 nm, corresponding to the (100) exposed crystal faces of CeO_2_ (Fig. 4(d)). The lattice spacing of nanoparticle CeO_2_ is 0.313 nm, aligning with the (111) exposed crystal face (Fig. 4(f)).^22,23^

In the case of nano spindle-shaped CeO_2_, the structure comprises densely packed nanocrystals, indicative of a polycrystalline nature. Observations reveal several light spots on the crystal surface, suggesting the presence of defects. The lattice spacing of 0.313 nm corresponds to the (111) exposed crystal faces of CeO_2_ (Fig. 4(h)). Significant disparities in crystal size and specific exposed crystal faces are evident among CeO_2_ morphologies.^23^ By modulating the growth of CeO_2_ along different crystal faces, the selectivity of CeO_2_ towards exposed crystal faces can be adjusted. This provides a basis for further exploration into the impact of CeO_2_ catalysts with varying morphologies on the catalytic oxidation of DCM.

**Fig. 5:**
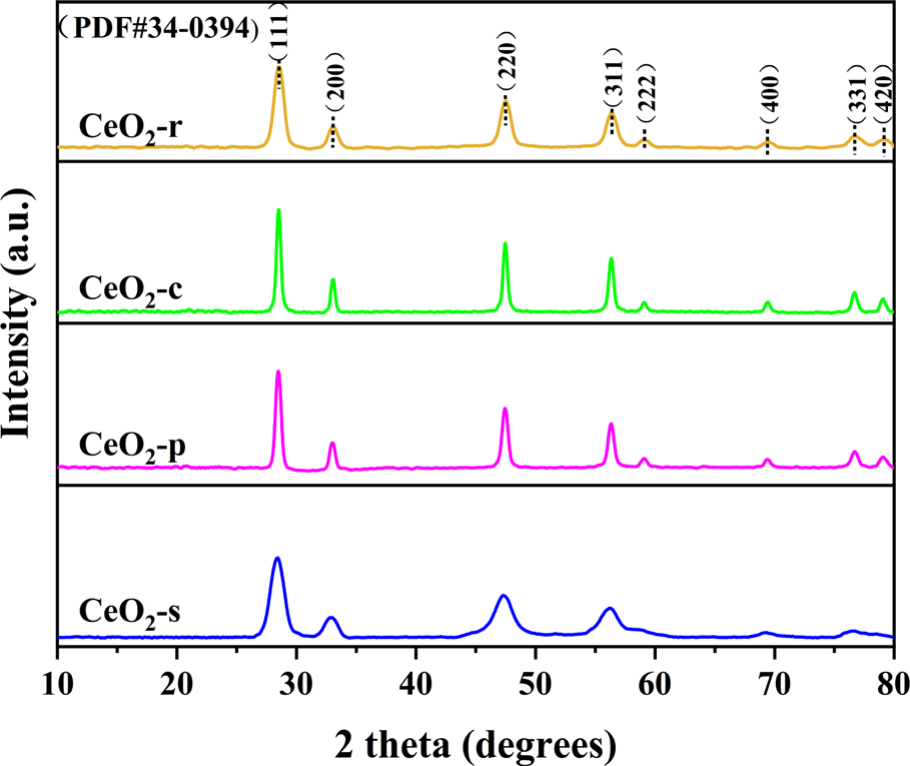
XRD patterns of CeO_2_ catalysts with different nanomorphologies.

The crystal structures of the four distinct CeO_2_ catalyst morphologies were meticulously examined through XRD mapping, as illustrated in Fig. 5. Characteristic diffraction peaks manifest at 2*θ* angles of 28.5°, 33.1°, 47.4°, 56.4°, 59.0°, 69.5°, 76.9°, and 79.1° for CeO_2_ catalysts with different morphologies. Comparative analysis with the standard card XRD (JCPDS PDF#34-0394) reveals that these diffraction peaks align with (111), (200), (220), (311), (222), (400), (331), and (420) exposed crystal faces, respectively. This unequivocally confirms that the four distinct CeO_2_ morphologies prepared exhibit a typical cubic fluorite structure with a space group of *Fm*3*m* and a cell parameter of *α* = 5.411 Å.^24^ Both the intensity and width of diffraction peaks are intimately linked to the material's degree of crystallinity. Peak intensity correlates positively with crystallinity, while peak width correlates negatively.^25^ Analyzing characteristic diffraction peak intensities and peak half-maximum full widths for all catalyst samples reveals a specific order: CeO_2_-c > CeO_2_-p > CeO_2_-r > CeO_2_-s for peak intensity, and CeO_2_-s > CeO_2_-r > CeO_2_-p > CeO_2_-c for peak width. Notably, CeO_2_-s exhibits the lowest diffraction peak intensity and the highest peak width among the various CeO_2_ morphologies, signifying relatively low crystallinity and a propensity for lattice defects. This finding aligns consistently with the SEM and TEM results. By applying Scherrer's formula to the full width at half maximum of the diffraction peak at 2*θ* = 28.5°, the grain sizes of CeO_2_ with different morphologies were calculated. The resulting order of grain sizes is CeO_2_-c (26.47 nm) > CeO_2_-p (22.08 nm) > CeO_2_-r (12.06 nm) > CeO_2_-s (8.82 nm), with spindle-shaped CeO_2_ boasting the smallest grain size. The grain size of CeO_2_ significantly influences the content of surface oxygen species and the concentration of defective oxygen vacancies.^26^ Smaller grain size CeO_2_ catalysts are advantageous for exposing surface-active sites, thereby exhibiting heightened catalytic degradation effects.

**Fig. 6:**
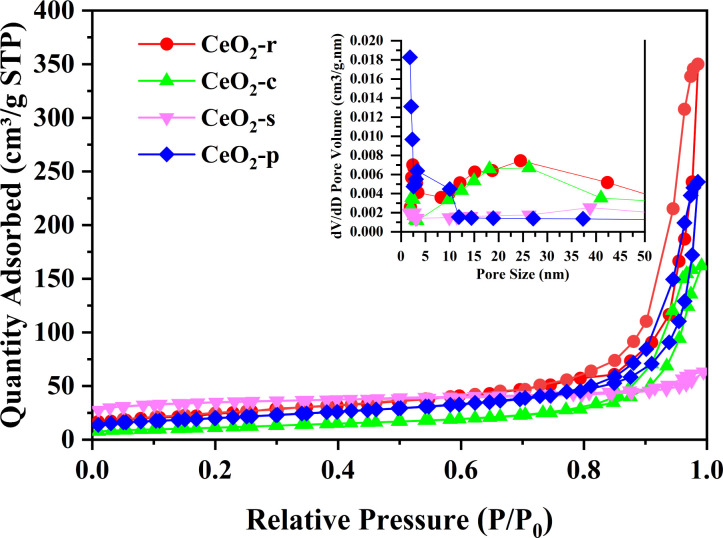
Nitrogen adsorption–desorption isotherms of CeO_2_-x (x = r, c, p, s), and the corresponding BJH pore size distribution.

The N_2_ adsorption–desorption isotherms and BJH pore size distribution for CeO_2_ with various morphologies are elucidated in Fig. 6. The adsorption–desorption isotherms of the four CeO_2_ catalyst morphologies showcase typical H3-type hysteresis loops within the *P*/*P*_0_ range of 0.4 to 0.97. Following IUPAC classification, the isotherms for all CeO_2_ catalysts, regardless of morphology, fall under type IV, signifying narrow mesoporous structures for each.^27^ The starting height of the hysteresis loop is proportionate to the specific surface area of the samples.^28^ Notably, the loop starting heights follow the order: CeO_2_-s > CeO_2_-r > CeO_2_-p > CeO_2_-c. This implies that CeO_2_-s boasts the largest specific surface area, aligning consistently with the findings presented in Table 1. Concurrently, the pore size distribution (BJH) of CeO_2_ catalysts is depicted in the figure, revealing varying but mesoporous structures for all morphologies. Specific surface area, pore volume, and pore size for the four CeO_2_ catalysts are detailed in Table 1 Notably, the specific surface area of CeO_2_-s stands at 107.8 m^2^ g^−1^, significantly surpassing CeO_2_-r (85.9 m^2^ g^−1^), CeO_2_-c (40.5 m^2^ g^−1^), and CeO_2_-p (70.6 m^2^ g^−1^). Tamboli *et al.*^29^ similarly prepared four different shapes of CeO_2_, namely sphere, mixed shape, spindles, and rod, where CeO_2_ spindles exhibited the largest specific surface area (104 m^2^ g^−1^), which is consistent with the results tested here. A larger specific surface area facilitates increased exposure of active sites, thereby enhancing pollutant adsorption on the catalyst surface and promoting more thorough oxidative decomposition.^30^ Pore size analysis indicates similar sizes for CeO_2_-r (20.7 m^2^ g^−1^), CeO_2_-c (23.2 nm), and CeO_2_-p (27.9 nm) catalysts, while CeO_2_-s exhibits a markedly smaller pore size of 3.4 nm. A smaller pore size suggests poorer crystallization effects, rendering the material prone to lattice defects and subsequently elevating the surface concentration of oxygen vacancies. This observation aligns with the combined analysis of SEM and XRD characterizations.

**Table 1:** Specific surface area, pore volume, pore size and grain size of CeO_2_ catalysts with different nanomorphologies

Samples	*S*_BET_ (m^2^ g^−1^)	Pore volume (cm^3^ g^−1^)	Pore size (nm)	Grain size (nm)
CeO_2_-r	85.9	0.365	20.7	12.06
CeO_2_-c	40.5	0.235	23.2	26.47
CeO_2_-p	70.6	0.108	27.9	22.08
CeO_2_-s	107.8	0.091	3.4	8.82

### 3.2 Surface chemical states

**Fig. 7:**
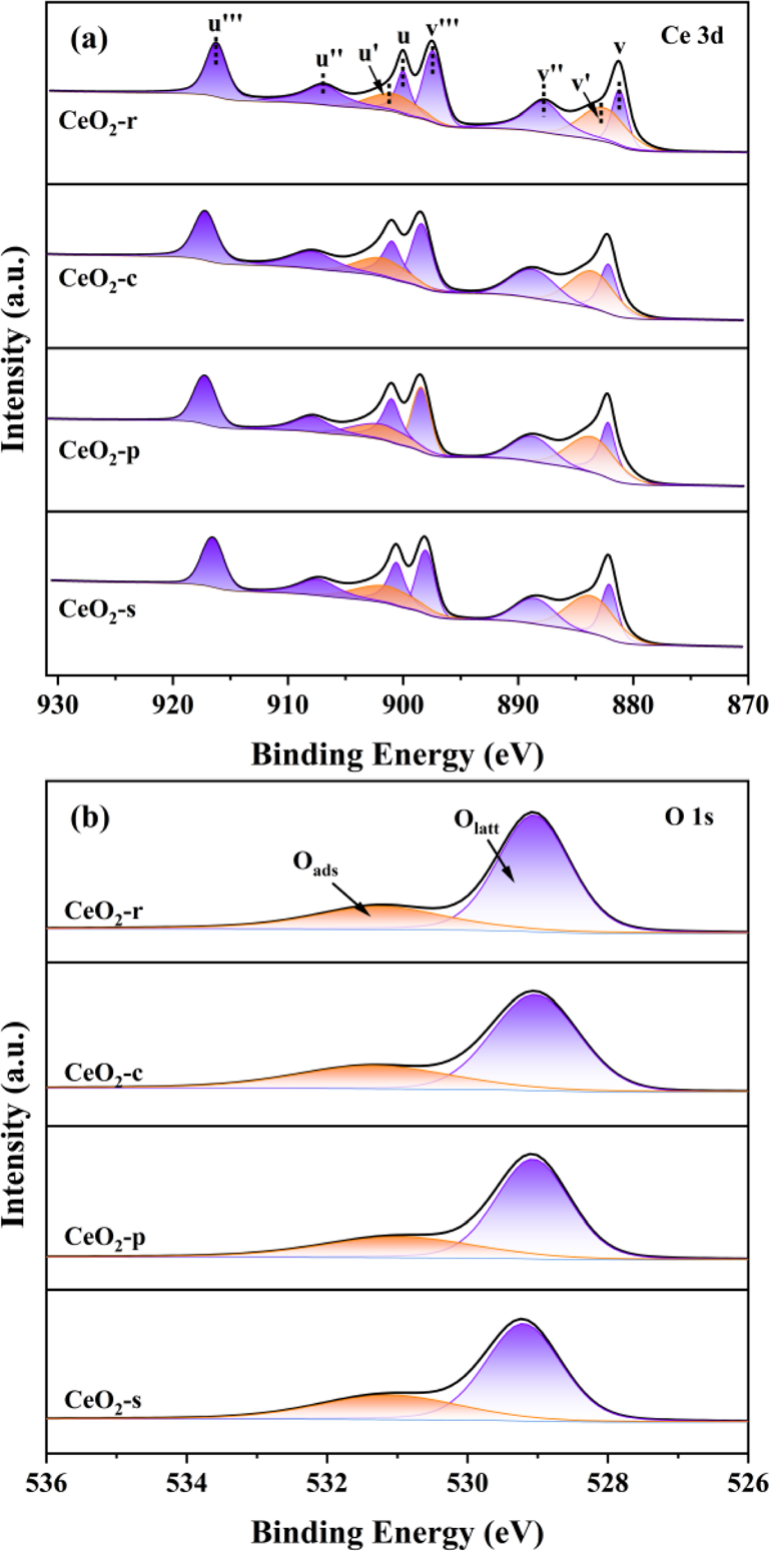
XPS patterns of Ce 3d (a) and O 1s (b) for different nanomorphologie of CeO_2_ catalysts.

X-ray Photoelectron Spectroscopy (XPS) serves to elucidate the electronic layering of atoms or molecules present on the catalyst surface, facilitating a comprehensive analysis of surface elemental composition and valence states within the catalyst specimen. In Fig. 7(a), the Ce 3d photoelectron spectra of CeO_2_ catalysts with diverse morphologies are depicted. It is noteworthy, as elucidated in the pertinent literature,^31^ that the deconvolution process resulted in the identification of eight distinct sets of peaks for Ce 3d, encompassing two spin orbitals, namely 3d_5/2_ (u) and 3d_3/2_ (v). This finding signifies the coexistence of two valence states, Ce^4+^and Ce^3+^, on the surfaces of CeO_2_ catalysts with varying morphologies.

The distinctive peaks in the Ce 3d spectrum, denoted as *u* (900.8 eV), *u*′′ (907.2 eV), *u*′′ (816.6 eV), *v* (882.3 eV), *v*′′ (888.6 eV), and *v*′′ (897.7 eV), are unequivocally associated with Ce^4+^. In contrast, the characteristic peaks located at *u*′ (902.5 eV) and *v*′ (884.9 eV) correspond to Ce^3+^. This phenomenon entails the relinquishment of electrons by lattice oxygen atoms, leading to their departure from the lattice site and subsequently giving rise to the generation of oxygen vacancies.

Simultaneously, Ce^4+^ undergoes electron acquisition, converting into Ce^3+^, thereby signifying the occurrence of oxygen vacancy generation. The presence of Ce^3+^ serves as an indicator, and its concentration exhibits a positive correlation with the oxygen vacancy concentration.^32^ Oxygen vacancies on the catalyst surface facilitate the adsorption of gas-phase oxygen, and a sequential migration process occurs as follows: O_2_ → 2O → O_2_^−^ → 2O^−^ → 2O^2−^.^33^ This migration mechanism promotes the catalytic oxidation of DCM. To semi-quantitatively assess the ratio of Ce^3+^ to total Ce in different orbitals, calculations were based on the characteristic peak area ratio of Ce^3+^ to total Ce. As outlined in Table 2, CeO_2_-s exhibits the highest Ce^3+^ content across various orbitals. This observation may be attributed to the nano spindle-shaped CeO_2_, as revealed in transmission electron microscopy (TEM), featuring a concave–convex interface resulting from extensive planar interlacing. The elevated Ce^3+^ content indicates a higher concentration of oxygen vacancies in this morphology, which, in turn, adsorb and activate gas-phase oxygen, transforming into highly active surface oxygen species. This process promotes their migration, thereby enhancing the catalytic oxidation performance of DCM. Conversely, CeO_2_-c demonstrates a lower Ce^3+^ content compared to the other three morphologies. This observation aligns with the crystallographic analysis using scanning electron microscopy (SEM) and X-ray diffraction (XRD), suggesting that the nano cubic-shaped CeO_2_ possesses a complete and smoothly oriented surface crystal structure.

**Table 2:** XPS results of CeO_2_ catalysts with different nanomorphologies

Samples	3d_3/2_ Ce^3+^	Ce 3d 3d_5/2_ Ce^3+^	Ce^3+^/(Ce^3+^ + Ce^4+^) (%)	O 1s O_ads_/(O_latt_ + O_ads_) (%)
CeO_2_-r	22.51	32.41	28.1	31.63
CeO_2_-c	20.77	27.97	24.82	28.71
CeO_2_-p	21.13	30.53	26.33	32.31
CeO_2_-s	25.51	35.84	31.15	35.18

Fig. 7(b) presents the O 1s photoelectron spectroscopy results for four distinct morphologies of CeO_2_. Based on the binding energy, these spectra are categorized into two groups of characteristic peaks. Peaks occurring around 529.4 eV are attributed to lattice oxygen (O_latt_), those at approximately 531.4 eV correspond to surface adsorbed oxygen (O_ads_).^34,35^ Notably, Oads encompasses O_2_^−^ and O_2_^2−^, serving as active oxygen species generated on oxygen vacancies, effectively catalyzing the degradation of adsorbed DCM. Consequently, the ratio of O_ads_/(O_latt_ + O_ads_) serves as an indicator of O_ads_ concentration and provides an assessment of oxygen vacancy concentration.^36^ Quantitative analysis of characteristic peak areas, as detailed in Table 2, reveals the following order for the O_ads_/(O_latt_ + O_ads_) ratio: CeO_2_-s (35.18%) > CeO_2_-p (32.31%) > CeO_2_-r (31.63%) > CeO_2_-c (28.71%). CeO_2_-s exhibits a notably higher ratio, indicating a more substantial concentration of surface adsorbed oxygen compared to other CeO_2_ morphologies. This finding further substantiates that the nano spindle-shaped CeO_2_ surface features the highest oxygen vacancy concentration, a conclusion consistent with the Ce 3d spectroscopy results.

**Fig. 8:**
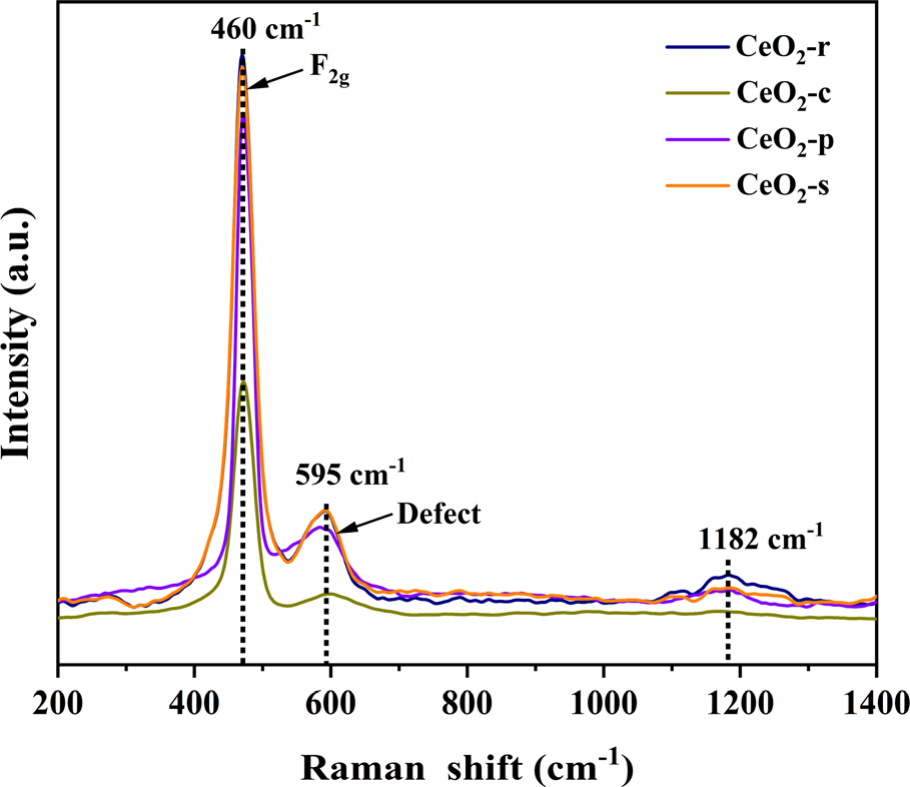
Raman spectra of CeO_2_ catalysts with different nanomorphologies.

The molecular structure of the prepared catalysts was scrutinized through Raman spectroscopy, and the outcomes of Raman spectroscopic characterization for CeO_2_ with distinct morphologies are presented in Fig. 8 All four diverse nanomorphologies of CeO_2_ catalysts exhibit prominent characteristic peaks at 460, 595, and 1182 cm^−1^. The Raman peak observed at 460 cm^−1^ corresponds to the symmetric telescopic vibrational peaks (F_2g_) associated with the Ce^4+^ cation and the surrounding eight O^2−^ anions in the cubic fluorite structure of CeO_2_. This observation corroborates the cubic crystalline structure of CeO_2_, aligning with the X-ray diffraction (XRD) results.^37^ The Raman peak at 595 cm^−1^ is attributed to the characteristic peak of oxygen vacancy, specifically the Frenkel defect induction mode (D) induced by the presence of Ce^3+^.^38^ Additionally, the Raman peak at 1182 cm^−1^ corresponds to the second-order longitudinal optical vibration peak (2LO).^39^ In general, the ratio of peak D to peak F_2g_ intensity (*I*_D_/*I*_F_2g__) allows for the quantitative analysis of the surface oxygen vacancy concentration in CeO_2_. A higher ratio indicates a greater surface oxygen vacancy concentration.^40,41^ Oxygen vacancies, as significant structural defects in metal oxides, serve as a pivotal reference index for evaluating catalytic oxidation performance. They not only adsorb gas-phase oxygen molecules, forming highly reactive surface oxygen species in catalytic reactions, but also directly act as adsorption sites for pollutants, enhancing catalytic degradation effectiveness. The *I*_D_/*I*_F_2g__ ratios for the four distinct nanomorphologies of CeO_2_ catalysts follow the order: CeO_2_-s > CeO_2_-r > CeO_2_-p > CeO_2_-c. This sequence further affirms that the relatively high concentration of oxygen vacancies on the surface of nano spindle-shaped CeO_2_ is conducive to the catalytic oxidation of DCM, a conclusion consistent with the X-ray photoelectron spectroscopy (XPS) results.

### 3.3 Temperature programmed characterizations

**Fig. 9:**
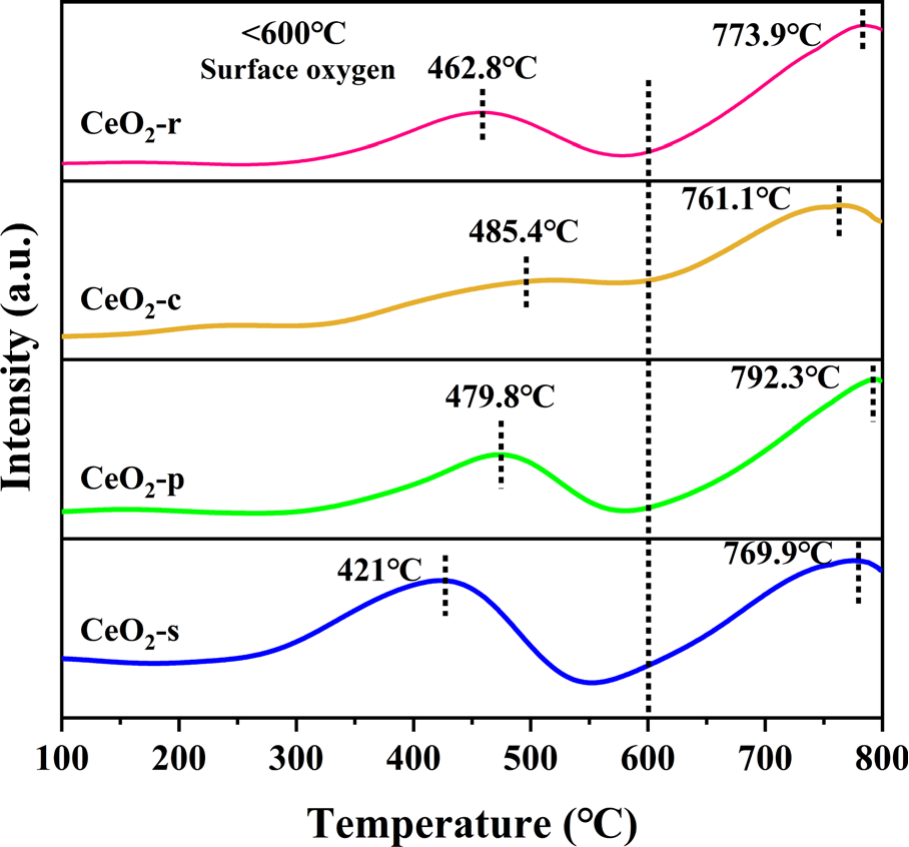
H_2_-TPR of CeO_2_ with different nanomorphologies.

CeO_2_, owing to its facile interconversion between Ce^3+^ and Ce^4+^, possesses exceptional redox properties and finds widespread applications in the field of catalytic oxidation. The low-temperature reduction properties of four distinct nanomorphologies of CeO_2_ were investigated using H_2_-TPR spectroscopy. Typically, the reduction of CeO_2_ can be categorized into three temperature intervals, namely surface oxygen reduction at 250–400 °C, subsurface oxygen reduction at 400–600 °C, and bulk oxygen reduction at 600–1000 °C.^42^ As depicted in Fig. 9, all four diverse nanomorphologies of CeO_2_ exhibit two sets of high-resolution reduction peaks. The reduction peak at <600 °C corresponds to the reduction of CeO_2_ surface oxygen, whereas the peak at >600 °C signifies the reduction of CeO_2_ bulk-phase oxygen.^43^ The primary focus centers on the investigation of the reduction peak in the low-temperature range (<600 °C), as it directly reflects the extent of involvement of surface-active oxygen species and is intricately linked to the catalytic performance at low temperatures.

Evident from the sequence of reduction peak signals, the temperatures at which the reduction peaks occur follow the order: CeO_2_-c > CeO_2_-p > CeO_2_-r > CeO_2_-s. CeO_2_-c exhibits a reduction peak at 485.4 °C, while the reduction peaks for other CeO_2_ morphologies shift towards lower temperatures. Specifically, CeO_2_-s registers the lowest peak temperature, with a maximum temperature difference of 64.4 °C compared to CeO_2_-c. Generally, a lower temperature for the occurrence of the reduction peak signifies superior redox performance. Robust redox properties are advantageous for the catalytic oxidation of DCM.^44–46^ This observation suggests that the surface oxygen species on the nano spindle-shaped CeO_2_ catalyst are more active, demonstrating enhanced low-temperature reduction performance. To delve deeper into the reduction performance of the prepared catalyst, hydrogen consumption was analyzed. Based on the peak area of the hydrogen consumed by reduction, the chemisorbent for the test had the peak area of the corresponding hydrogen consumption calibrated, which led to the calculation of the hydrogen consumption during the TPR of different samples.^47,48^

As shown in Table 3, In the low-temperature interval, the hydrogen consumption of CeO_2_ with different nanomorphologies follows the order: CeO_2_-s > CeO_2_-r > CeO_2_-p > CeO_2_-c, with the hydrogen consumption of CeO_2_-s being 2.13 times that of CeO_2_-c. It is well-established that hydrogen consumption is directly proportional to the content of surface-active oxygen species.^49,50^ The hydrogen consumption results further indicates that the spindle-shaped CeO_2_ catalysts, featuring exposed crystal faces of (111) are abundant in surface-active oxygen species. Consequently, CeO_2_-s exhibit excellent low-temperature reduction properties and hold significant potential for catalyzing the oxidation of DCM. Notably, this finding aligns with the X-ray photoelectron spectroscopy (XPS) results.

**Table 3:** Hydrogen consumption of CeO_2_ with different nanomorphologies

Samples	Peak position (°C)	H_2_ consumption (mmol g^−1^)
CeO_2_-r	462.8	0.673
CeO_2_-c	485.4	0.412
CeO_2_-p	479.8	0.614
CeO_2_-s	421	0.878

### 3.4 Evaluation of catalyst activity

**Fig. 10:**
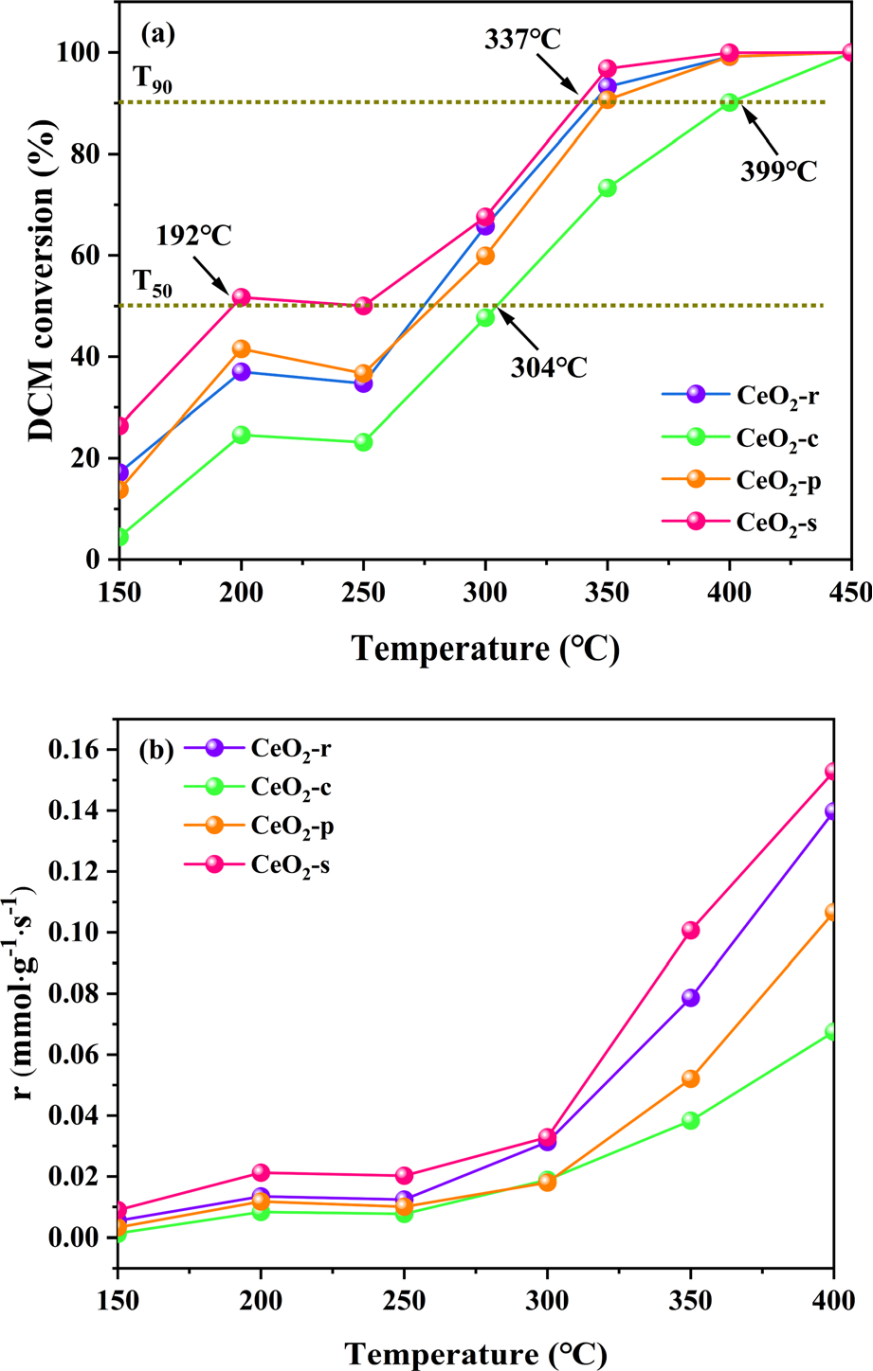
DCM catalytic performance (a) and reaction rate (b) of CeO_2_ with different nanomorphology.

The catalytic degradation effects on DCM by the four prepared catalysts with different nanomorphologies (CeO_2_-r, CeO_2_-c, CeO_2_-p, CeO_2_-s) in the temperature interval of 150–450 °C are illustrated in Fig. 10. The corresponding temperatures (*T*_50_, *T*_90_), representing the achievement of 50% and 90% conversion rates of DCM over the different morphologies of CeO_2_ catalysts, are presented in Table 4. As depicted in Fig. 10(a), the conversion rate of DCM over the four different nanomorphologies of CeO_2_ catalysts exhibits an ascending trend with increasing temperature. All catalyst samples demonstrate complete catalysis of DCM oxidation at temperatures up to 450 °C. However, during the programmed temperature increase (<450 °C), the catalytic oxidation effects of different nanomorphologies of CeO_2_ on DCM vary significantly. The order of reaching *T*_50_ is as follows: CeO_2_-c (304 °C) > CeO_2_-p (277 °C) > CeO_2_-r (268 °C) > CeO_2_-s (192 °C), with CeO_2_-s achieving *T*_50_ at less than 200 °C. This temperature is significantly lower than the other three morphologies, and when compared with CeO_2_-c, the difference between the two-Δ*T*_50_ = 112 °C. This result suggests that the nano spindle-shaped CeO_2_ exhibits superior catalytic activity at low temperatures.

**Table 4:** Catalytic activity of CeO_2_ catalysts with different nanomorphologies

Samples	*T*_50_ (°C)	*T*_90_ (°C)
CeO_2_-r	268	344
CeO_2_-c	304	399
CeO_2_-p	277	347
CeO_2_-s	192	337

The order in which different nanomorphologies of CeO_2_ reach *T*_90_ is as follows: CeO_2_-c (399 °C) > CeO_2_-p (347 °C) > CeO_2_-r (344 °C) > CeO_2_-s (337 °C), with CeO_2_-s achieving *T*_90_ at the lowest temperature, exhibiting a difference of Δ*T*_90_ = 62 °C compared to CeO_2_-c. The nano spindle-shaped CeO_2_ catalysts results show that featuring exposed crystal faces of (111) is significantly robust catalytic degradation effects on DCM. Considering the aforementioned characterization results, the nano spindle-shaped CeO_2_ catalysts, in comparison with the other three morphologies of CeO_2_ catalysts, exhibit characteristics such as a large specific surface area, small pore size, weak crystallinity, robust low-temperature reduction performance, high oxygen vacancy concentration, and abundant surface oxygen species. These characterization results further affirm that nano spindle-shaped CeO_2_ catalysts exert a more pronounced catalytic oxidation effect on DCM.

In Fig. 10(a), all four CeO_2_ catalysts with different morphologies exhibit a transient deactivation phenomenon in the temperature interval of 200–250 °C. The conversion rates of DCM decrease to varying degrees with the rise in temperature, a behavior that may be attributed to the strong adsorption of chlorine (Cl) species from DCM at lower temperatures onto active sites on the catalyst surface.^51,52^ As the reaction temperature increases and oxygen diffuses through the surface lattice, the Cl species adsorbed on the catalyst surface gradually desorb, leading to a restoration of the activities of the various CeO_2_ catalyst morphologies, with an increasing trend.

Fig. 10(b) shows the reaction rates of CeO_2_ catalysts with different morphologies at 150–400 °C, the order in which different nanomorphologies of reaction rates are as follows: CeO_2_-s > CeO_2_-r > CeO_2_-p > CeO_2_-c. Which indicates that CeO_2_-s has the highest reaction rate for the most efficient conversion of DCM as the temperature increases, and this result is in agreement with Fig. 10(a).

### 3.5 Product distribution of catalysts

**Fig. 11:**
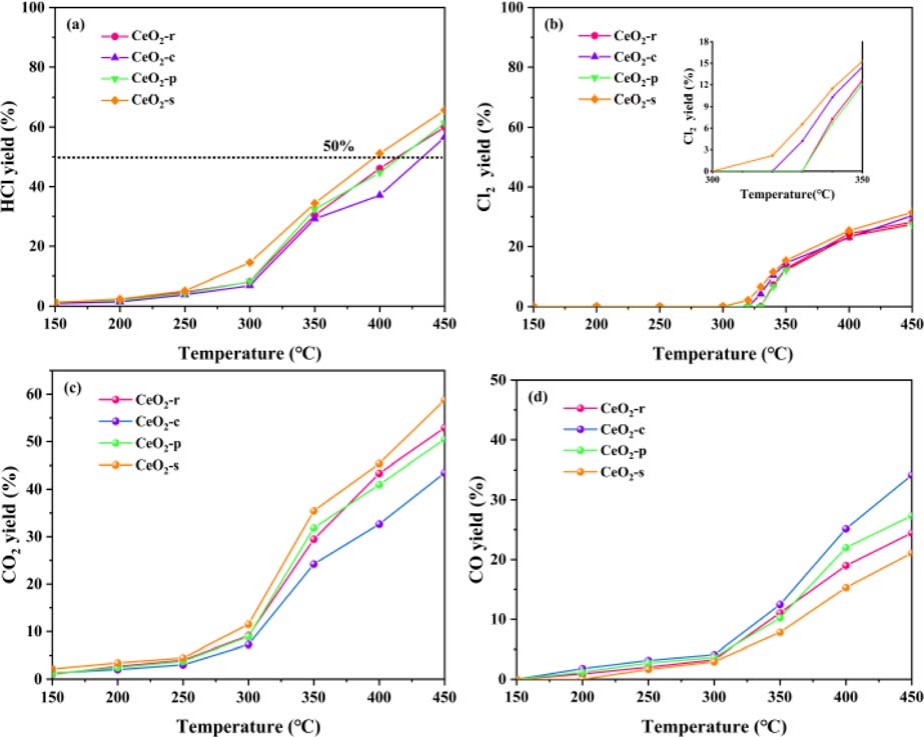
Distribution of HCl (a), Cl_2_ (b), CO_2_ (c), CO (d) yields of CeO_2_ with different nanomorphologies at different temperatures.

The nature of a catalyst plays a crucial role in determining its product distribution. To investigate the correlation between exposed crystal faces and product selectivity, CeO_2_ nanomorphologies were deliberately controlled by altering the preparation conditions. This regulation aimed to influence the selectivity towards exposed crystal faces during the catalytic oxidation of DCM using different morphologies of CeO_2_. The catalytic products, including HCl, Cl_2_, CO_2_, and CO in the exhaust gas, were collected, and the yields were calculated and analyzed using the provided equation. The HCl yield of the catalytic products from the four distinct nanomorphologies of CeO_2_ catalysts is illustrated in Fig. 11(a). It is evident that all catalysts exhibit an increasing trend in HCl yield within the temperature interval of 150–450 °C. The yield follows the order: CeO_2_-s > CeO_2_-p > CeO_2_-r > CeO_2_-c. The prepared catalysts achieve yields exceeding 50%, indicating robust HCl selectivity. Evidently, the nano spindle-shaped CeO_2_ catalysts with exposed crystal faces (111) demonstrate the highest HCl yield at 65.67%, underscoring their superior performance in this regard.

The HCl yield of CeO_2_ catalysts, across all samples, exhibits a gradual increase in the temperature interval of 150–250 °C. During this interval, the HCl yield for all catalysts remains below 10%, potentially attributed to the fact that HCl does not reach its desorption temperature before 250 °C. As the temperature rises to 300 °C, the incline of the HCl yield curves for all catalysts notably intensifies. The distribution of Cl_2_ yield for different morphologies of CeO_2_ catalysts is depicted in Fig. 11(b). All catalysts generate a certain amount of Cl_2_ at higher temperatures (>300 °C), a phenomenon that may be associated with the Deacon reaction (4HCl + O_2_ → 2Cl_2_ + 2H_2_O). CeO_2_-s exhibits a notably high Cl_2_ yield. In conjunction with X-ray photoelectron spectroscopy (XPS) and Raman characterization results, CeO_2_-s demonstrates a higher oxygen vacancy concentration than other morphologies. This characteristic promotes the migration of surface lattice oxygen, facilitating the diffusion of lattice oxygen to the outer surface of the catalyst. This process replaces Cl species on the oxygen vacancy, thereby contributing to the generation of Cl_2_. Additionally, the Cl_2_ generation temperature of CeO_2_-s is 315 °C, lower than that of the other three morphologies. This is attributed to the richer content of surface oxygen species on the surface of the CeO_2_-s catalyst. The Cl_2_ generation temperature of CeO_2_-p is observed to be 335 °C, indicating an increased temperature for Cl_2_ generation.

The yields of CO_2_ and CO for all catalyst samples are depicted in Fig. 11(c and d), offering insight into the redox properties by comparing the CO_*x*_ yield of CeO_2_ with different morphologies. The CO_*x*_ yield for all four nanomorphologies of CeO_2_ increases with temperature, yet their yields differ. Specifically, CeO_2_-s exhibits the highest CO_2_ yield, CeO_2_-r and CeO_2_-p demonstrate similar yields, while the CO_2_ yield of CeO_2_-c is notably lower than the three aforementioned morphologies. These results indicate that nano spindle-shaped CeO_2_ catalysts with exposed crystal faces of (111) possess superior redox capacity. Furthermore, the CO yield of all catalysts was analyzed Fig. 11(d), revealing that CeO_2_-c has a higher CO content than the other three morphologies. This suggests that the lower redox capacity of cubic CeO_2_ itself results in an inability to efficiently and swiftly oxidize intermediate transition products and CO generated during the reaction process to CO_2_, leading to a higher CO yield.

### 3.6 Stability and durability of catalysts

**Fig. 12:**
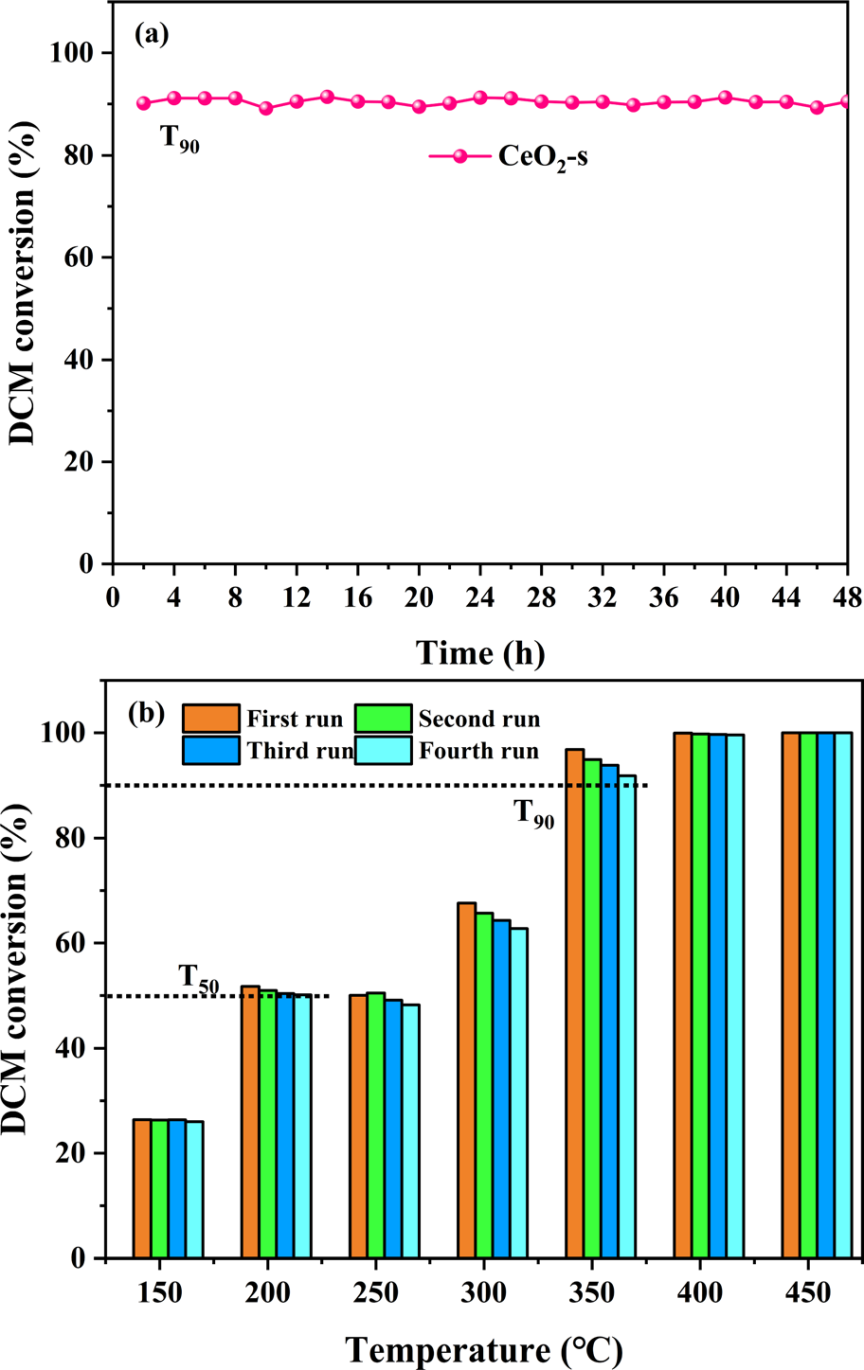
Stability performance test of CeO_2_-s catalyst for DCM catalytic oxidation (a), durability test of CeO_2_-s catalyst for DCM catalytic oxidation at 150–450 °C (b).

The long-term stability and reusability of CeO_2_-s in the catalytic oxidation of DCM were examined. The catalytic degradation rate of DCM by CeO_2_-s, operating continuously at a constant temperature of 340 °C for 48 hours, is illustrated in Fig. 12(a). The results indicate that there is no significant activity loss of the catalyst during the experiment, and the degradation rate of DCM remains stable at about 90% until the end of the experiment. This outcome suggests that CeO_2_-s exhibits good chlorine resistance and resistance to carbon accumulation, demonstrating stability in the catalytic oxidation of DCM. Additionally, four consecutive cycle tests were conducted on CeO_2_-s, and the degradation effect of DCM within the temperature interval of 150–450 °C is presented in Fig. 12(b). Throughout the continuous cycle test, the catalytic activity of CeO_2_-s shows a slight decreasing trend, but this has minimal impact on the overall catalytic reaction. In the four cycles, CeO_2_-s achieves *T*_50_ for DCM catalytic oxidation before 200 °C and *T*_90_ before 350 °C. This outcome further emphasizes the good stability of nano spindle-shaped CeO_2_, making it suitable for reuse in practical catalytic DCM applications and contributing to reduced treatment costs.

### 3.7 Effect of WHSV

**Fig. 13:**
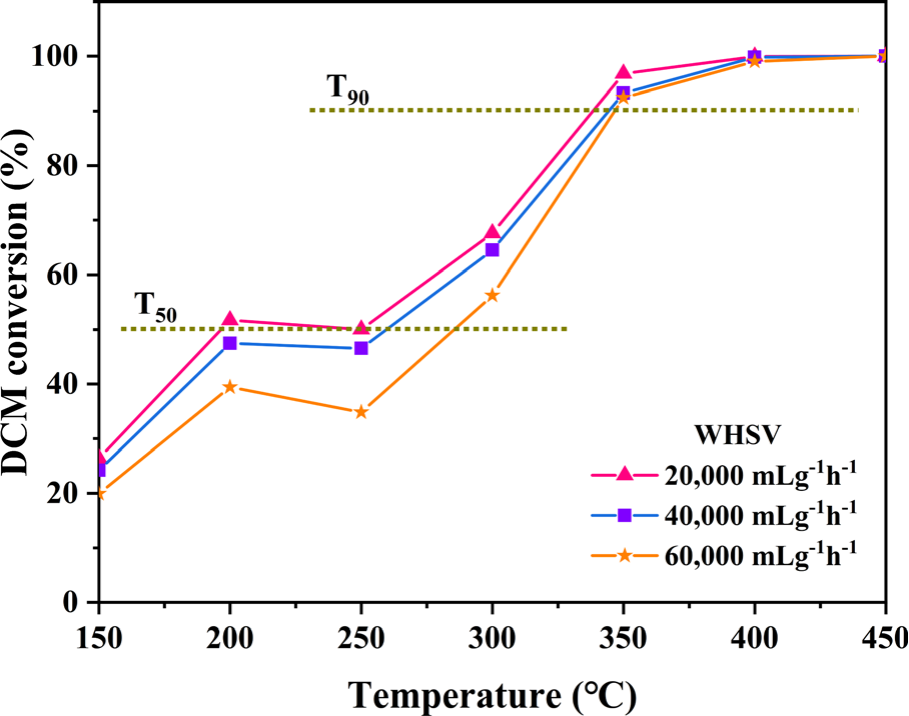
Effect of different WHSV on the DCM reaction performance of CeO_2_-s catalysts.

The impact of various airspeed conditions (Weight Hourly Space Velocity-WHSV) on the degradation of DCM over CeO_2_-s catalysts was investigated, as illustrated in Fig. 13. As the WHSV increases from 20 000 mL g^−1^ h^−1^ to 60 000 mL g^−1^ h^−1^, a decreasing trend is observed in the activity of CeO_2_-s catalysts. However, catalysts at different WHSV levels demonstrate the ability to achieve *T*_90_ at 350 °C against DCM, indicating enhanced resistance to DCM impact for nano spindle-shaped CeO_2_ catalysts. Specifically, at WHSV = 20 000 mL g^−1^ h^−1^, *T*_50_ and *T*_90_ are 192 °C and 337 °C, respectively, marking a decrease of 73 °C and 11 °C compared to WHSV = 60 000 mL g^−1^ h^−1^. These results suggest that at lower WHSV, DCM exhibits a prolonged residence time on the CeO_2_-s catalyst surface compared to higher WHSV conditions. This prolonged exposure facilitates the adsorption of DCM on the catalyst surface for activation, thereby promoting the catalytic degradation of DCM.

### 3.8 Water resistance of catalysts

**Fig. 14:**
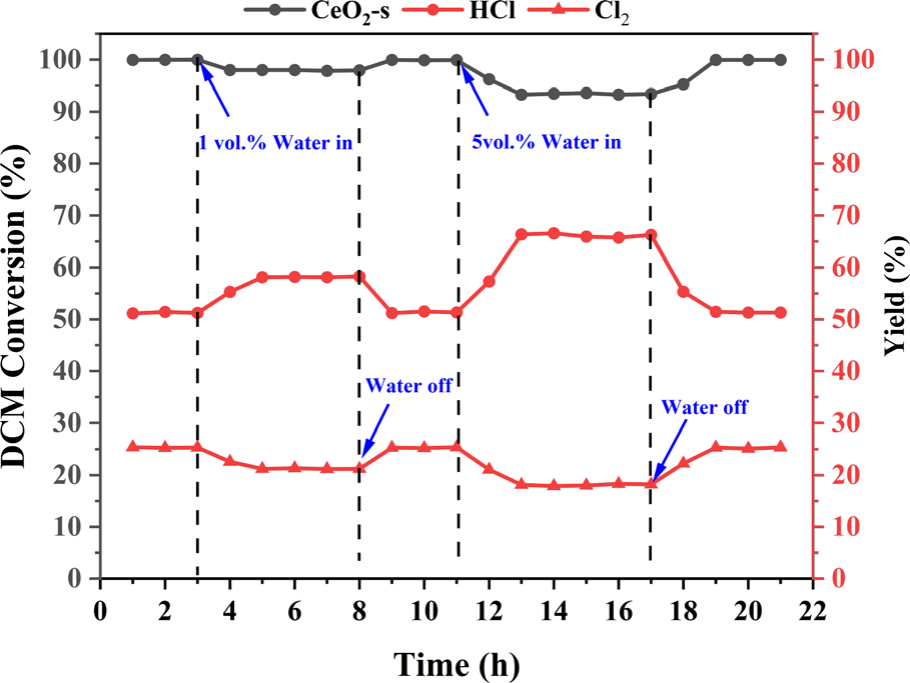
Effect of 1 vol% and 5 vol% H_2_O on DCM reaction performance over CeO_2_-s catalysts at 400 °C.

CeO_2_-s catalysts demonstrate effectiveness in the catalytic oxidation of DCM under ideal drying conditions. However, typical industrial exhaust gases often contain approximately 5 vol% moisture, a factor that commonly hampers catalyst activity and influences the catalytic degradation efficiency.^53,54^ The water resistance of CeO_2_-s catalysts exhibiting optimal DCM catalytic oxidation performance was investigated by introducing 1 vol% and 5 vol% H_2_O into the mixed dry gas. As depicted in Fig. 14, at a constant temperature of 400 °C, the CeO_2_-s catalyst maintains stable activity with DCM conversion hovering around 99.5%. Upon the addition of 1 vol% H_2_O to the gas mixture, the CeO_2_-s catalyst experiences show a slight impact on DCM conversion, resulting in a decrease in DCM conversion to approximately 98%. Meanwhile, HCl production increased from 51.17% to 58.21% and Cl_2_ production decreased from 25.32% to 21.27%. Upon cessation of water vapor addition to the gas mixture, DCM conversion and HCl/Cl_2_ production gradually reverts to its initial level before reaching stability. Upon the addition of 5 vol% H_2_O to the gas mixture, the CeO_2_-s catalyst experiences a noticeable impact, resulting in a decrease in DCM conversion to approximately 93.29%. The results indicate that higher concentrations of water vapour cause slight catalyst deactivation. Despite the continuous introduction of a fixed amount of water vapor, DCM conversion remains stable at around 93%, underscoring the water resistance of nano spindle-shaped CeO_2_. Meanwhile, HCl production increased to 66.25% and Cl_2_ production decreased to 18.12%. Upon cessation of water vapor addition to the gas mixture, DCM conversion and HCl/Cl_2_ production gradually reverts to its initial level before reaching stability. The results indicate that water vapour in the gas mixture promotes the conversion and desorption of chlorine species adsorbed on the catalyst surface to HCl and inhibits the positive operation of the Deacon reaction, leading to an increase in the production of HCl and a decrease in the production of Cl_2_. At the same time, water molecules readily adsorb on the active sites of the catalyst surface, competing with DCM for adsorption. This competition renders the activation of DCM adsorption on the CeO_2_-s surface more challenging, consequently diminishing DCM conversion and relating with HCl/Cl_2_ production. However, it's noteworthy that the inhibition of catalyst activity by water vapor is of a physical nature, and this inhibition dissipates upon the removal of water vapor.

## 4 Conclusions

In this work, four CeO_2_ catalysts presented different catalytic performance, and the influence of exposed crystal surface was accordingly investigated. Compared to CeO_2_-r, CeO_2_-c and CeO_2_-p catalysts, CeO_2_-s catalysts exhibited the best DCM catalytic oxidation (*T*_90_ = 337 °C, *T*_50_ = 192 °C) and the higher HCl and CO_2_ production rates. Obvious characterizations included that CeO_2_-s catalysts owned smaller grain size, higher specific surface area, more surface oxygen species, higher concentration of oxygen vacancies, and low-temperature reductivity. Among them, smaller grain size and larger specific surface area contributed the most in the catalytic oxidation of DCM. Extensive performance tests were conducted on CeO_2_-s catalysts. Specifically, stability and durability tests suggested that CeO_2_-s exhibited robust stability and maintained consistently high activity over four usage cycles, showcasing excellent reusability and cost-effectiveness in practical applications. Water resistance and varying airspeed effect tests demonstrated that CeO_2_-s displayed efficient activity recovery after water vapor removal, while the catalytic activity for DCM performed a decreasing trend with increasing weight hourly space velocity.

It has been shown that the shape of CeO_2_ nanoparticles is influenced by multiple factors, including the nature of the solvent, the concentration of precursors, the use of additives, and the temperature and time of the reaction. The following are several factors that may influence the growth mechanism of CeO_2_ nanoparticles:

### 4.1 Solvent effect

The nature of the solvent (*e.g.* polarity, dielectric constant, *etc.*) can significantly affect the nucleation and growth process of nanoparticles. Heterogeneous solvents have different effects on the solubility of CeO_2_ precursors and the diffusion rate of reactants, thus changing the shape and size of particles.

### 4.2 Precursor concentration

The concentration of the precursor will affect the rate of nucleation and growth. Higher concentrations of precursors lead to rapid nucleation and the formation of smaller particles. The lower concentration may promote the growth of particles and form larger particles.

### 4.3 Additives

Additives or surfactants used in the synthesis process can be adsorbed onto specific crystalline surfaces, thereby affecting the direction and shape of particle growth. These molecules promote the formation of particles of specific shapes by selectively hindering the growth of certain crystalline surfaces.

### 4.4 Reaction temperature and time

Reaction temperature and time are crucial elements for the growth of CeO_2_ nanoparticles. Higher reaction temperatures generally accelerate nucleation and growth processes, while longer reaction times allow for further particle growth, which together determine the final shape and size of the particle.

### 4.5 Crystal growth kinetics

The growth of CeO_2_ nanoparticles is also affected by crystal growth kinetics. Crystal growth anisotropy (*i.e.*, differences in growth rates at different crystal planes) can lead to the formation of nanoparticles with diverse shapes.

## Conflicts of interest

The authors declare that they have no known competing financial interests or personal relationships that could have appeared to influence the work reported in this paper.

## Supplementary Material
